# The linearity of the El Niño teleconnection to the Amundsen Sea region

**DOI:** 10.1002/qj.3731

**Published:** 2020-02-03

**Authors:** Yu Yeung Scott Yiu, Amanda C. Maycock

**Affiliations:** ^1^ Centre for Atmospheric Sciences, Department of Chemistry University of Cambridge Cambridge UK; ^2^ School of Earth and Environment University of Leeds Leeds UK

**Keywords:** Amundsen Sea low, El Niño, ENSO, linearity, Rossby waves, teleconnection, West Antarctic climate

## Abstract

El Niño Southern Oscillation (ENSO) drives interannual variability in West Antarctic climate through altering atmospheric circulation in the Amundsen Sea region (ASR). The El Niño–ASR teleconnection is known to be strongest in austral winter and spring, but its variation with El Niño amplitude is underexplored. This study uses experiments from the HadGEM3‐A climate model to investigate the El Niño–ASR teleconnection for a range of imposed SST perturbations spanning weak (0.75 K) to strong (3 K) amplitudes. In austral winter, the El Niño–ASR teleconnection behaves linearly for El Niño amplitudes up to 2.25 K, but is found to weaken for stronger forcing (3 K). The anomalous Rossby wave source in the subtropical South Pacific increases monotonically with El Niño amplitude. However, a Rossby wave reflection surface originally located in the western South Pacific sector extends progressively eastward with increasing El Niño amplitude, reducing wave propagation into the ASR. The wave reflection surface is associated with curvature in the upper tropospheric zonal winds which intensifies as the subtropical jet strengthens under El Niño forcing. In contrast, the El Niño–ASR teleconnection in austral summer, which more closely resembles the Southern Annular Mode, is found to increase linearly for El Niño amplitudes up to 3 K. The results explicitly demonstrate that a linear approximation of the El Niño teleconnection to the ASR is reasonable based on the range of El Niño amplitudes observed in recent history.

## Graphical abstract

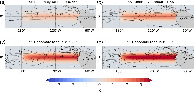

The tropical Pacific SST anomalies imposed in the four El Niño experiments (shading), which are held constant year round. Contours show the annual mean outgoing long‐wave radiation anomalies in the experiments. Contour interval is 20 W·m${}^{-2}$. Dotted contours show negative OLR anomalies.

## 1 Introduction

The Amundsen Sea low (ASL) is a quasi–stationary climatological low pressure centre found in the South Pacific sector of the Southern Ocean between the Antarctic Peninsula and the Ross Sea (Hosking *et al*., 2013; Turner *et al*., 2013; Raphael *et al*., 2016). As the ASL can be interpreted as the time average of synoptic and sub‐synoptic cyclones passing through the Amundsen Sea region (ASR; Fogt *et al*., 2012), the circumpolar storm tracks are important for the formation of the ASL. Indeed the existence of the ASL has been linked to zonal asymmetries in tropical sea surface temperatures (SSTs), which generate planetary‐scale Rossby waves that impact the Southern Hemisphere storm track (Inatsu and Hoskins, 2004), and the topography of Antarctica which causes asymmetries in synoptic activity (Lachlan‐Cope *et al*., 2001).

The ASL is characterized by a distinct seasonal cycle (Fogt *et al*., 2012; Hosking *et al*., 2013; Turner *et al*., 2013); it is deeper and located further southwest in austral winter (JJA) while it is weaker and further northeast in austral summer (DJF). The ASL exhibits some of the highest circulation variability in the Southern Hemisphere (Lachlan‐Cope *et al*., 2001) and has a strong impact on West Antarctic climate (Hosking *et al*., 2013; Turner *et al*., 2013; Raphael *et al*., 2016). Specifically, the ASL has been shown to impact surface wind, temperature, precipitation and sea ice concentrations in West Antarctica (Hosking *et al*., 2013) and the Ross Sea (Coggins and McDonald, 2015), and has been linked to long‐term trends in Antarctic sea ice (Turner *et al*., 2009; Turner *et al*., 2016). As a consequence, it is important to understand the factors that affect the behaviour of the ASL on interannual and longer time‐scales.

One phenomenon that greatly affects atmospheric circulation in the ASR is the El Niño Southern Oscillation (ENSO; Hoskins and Karoly, 1981; Karoly, 1989; Chen *et al*., 1996; Liu *et al*., 2002; Turner, 2004; Lachlan‐Cope and Connolley, 2006). Previous work has shown that the ASL is intensified during El Niño, and *vice versa* for La Niña (Bertler *et al*., 2004; Fogt *et al*., 2011; Turner *et al*., 2013). There is also a distinct seasonality to the ENSO–ASR teleconnection, being generally stronger in austral winter and spring and weaker in austral autumn and summer (Bertler *et al*., 2004; Jin and Kirtman, 2009; Turner *et al*., 2013; Yiu and Maycock, 2019).

Several mechanisms have been proposed to explain the ENSO teleconnection to the ASR, including its influence on the Pacific–South American (PSA) pattern (Hoskins and Karoly, 1981; Karoly, 1989; Schneider *et al*., 2011; Yiu and Maycock, 2019) and the Southern Annular Mode (SAM; Fogt and Bromwich, 2006; L'Heureux and Thompson, 2006; Fogt *et al*., 2011; Schneider *et al*., 2011). The PSA can be interpreted as a tropically forced Rossby wave train emanating from the tropical Pacific towards the ASR (Karoly, 1989). The ENSO–ASR teleconnection is strongest in austral winter (JJA), despite the fact that ENSO events tend to peak in austral spring to summer (Turner *et al*., 2013; Jin and Kirtman, 2009; Yiu and Maycock, 2019), and in this season the teleconnection exhibits a strong PSA‐like pattern (Karoly, 1989; Yiu and Maycock, 2019). In contrast, in austral summer the El Niño teleconnection at high southern latitudes more closely resembles the SAM (Yiu and Maycock, 2019).

The magnitude and spatial structure of observed El Niños vary substantially (Capotondi *et al*., 2015), but the extent to which these variations influence the teleconnection to the ASR is not well understood. Many previous studies have used meteorological reanalysis datasets to study the El Niño–ASR teleconnection (Kreutz *et al*., 2000; Bertler *et al*., 2004; Turner *et al*., 2013), but these datasets currently only encompass around 15 moderate to strong El Niños. As a consequence, studies often perform composite analyses that do not distinguish between the strength of El Niños and La Niñas (Fogt *et al*., 2011; Turner *et al*., 2013). Studies using composite and correlation analyses implicitly assume a linear relationship to ENSO amplitude (e.g., Bertler *et al*., 2004; Fogt *et al*., 2011), but to our knowledge this assumption has not been explicitly tested.

In this study, we investigate the linearity of the El Niño–ASR teleconnection using a set of climate model experiments that impose a fixed east Pacific El Niño SST anomaly of varying amplitude spanning weak (0.75 K) to strong (3 K) events. We investigate the dynamical mechanisms identified in previous studies (Hoskins and Karoly, 1981; Karoly, 1989; Schneider *et al*., 2011; Yiu and Maycock, 2019) to help explain the linearity (or lack thereof) in the simulated teleconnection to the ASR.

## 2 Data and methods

### 2.1 Model and experiment design

Experiments are performed with the Hadley Centre Global Environment Model version 3 (HadGEM3) (version 8.4 Global Atmosphere (GA) 4.0) with N96 horizontal resolution (1.875° longitude × 1.25° latitude) and 85 vertical levels up to an altitude of ∼84 km (Walters *et al*., 2014). The reference SSTs and sea ice prescribed in the model are taken from the HadISST dataset (Rayner *et al*., 2003). The control simulation uses prescribed monthly‐varying SSTs and sea ice fields averaged over 1995–2005 to represent approximate year 2000 climatological conditions that are close to an ENSO neutral state. All other boundary conditions (greenhouse gases, aerosols) represent year 2000 conditions and are held fixed in all experiments. Four time‐slice perturbation experiments are performed with SST anomalies imposed in the tropical Pacific to investigate the linearity of the ENSO–ASR teleconnection. Both the control and perturbed time‐slice experiments are 54 years long and the experiments are the same as those described by Trascasa‐Castro *et al*. (2019) and Yiu and Maycock (2019).

**Figure 1:**
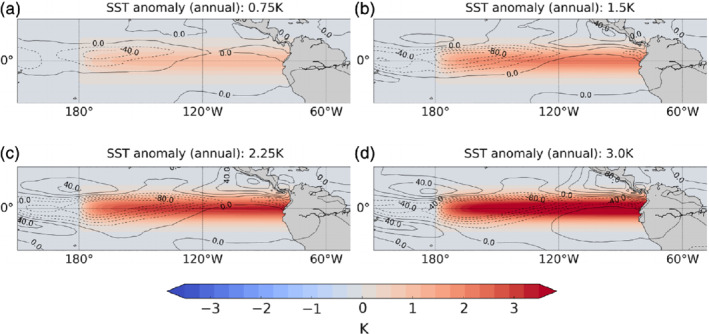
The tropical Pacific SST anomalies imposed in the four El Niño experiments (shading), which are held constant year round. Contours show the annual mean outgoing long‐wave (OLR) radiation anomalies in the experiments, with contour interval 20 W·m${}^{-2}$. Dotted contours show negative OLR anomalies [Colour figure can be viewed at wileyonlinelibrary.com]

The imposed SST perturbations are designed to capture the broad pattern of a classical east Pacific El Niño and do not attempt to mimic a specific real‐world event. We define the SST anomalies, δT(λ,ϕ), according to the function

(1) $$\delta T(\lambda ,\phi )=\left\{\begin{array}{c} \alpha \tan^{-1}\left(\frac{\lambda -180}{6}\right)\exp(-0.03\phi^{2})\\ \;\text{if}\;180^{\circ }\leq \lambda \leq 285^{\circ }\;\text{and –}10^{\circ }\leq \phi \leq 10^{\circ },\\ 0\;\text{otherwise}, \end{array}\right.$$

where α is a scaling factor and the other nomenclature is standard. In the four perturbation experiments, α is set to 0.58, 1.15, 1.73 and 2.30, which corresponds to Niño 3.4 index anomalies of 0.75, 1.5, 2.25 and 3.0 K, respectively (Figure 1). The pattern correlation over the region 180°E–270°E, 10°N–10°S between the imposed SST anomaly and an observed El Niño SST composite for DJF is 0.76; however, for individual El Niño events the pattern correlation in DJF is as high as 0.83 for the 2015/16 event. Also shown in Figure 1 are the simulated annual mean outgoing long‐wave radiation (OLR) anomalies in the tropical Pacific corresponding to each SST perturbation. This shows that convection in the Pacific intensifies and shifts towards the central Pacific under increasing El Niño forcing (also Trascasa‐Castro *et al*., 2019), which is in agreement with the observed response to east Pacific El Niños (Johnson and Kosaka, 2016).

The imposed SST anomalies are held constant throughout the 54 years of each experiment. This is unrealistic because observed El Niños evolve over the seasonal cycle, generally being strongest in austral summer and weakest in austral winter. The 3.0 K anomaly is around twice the amplitude of the strongest Niño 3.4 anomaly observed in JJA over the period 1870–2018, but is comparable to the strongest event observed in DJF (Supporting Information Figure S1). Hence the amplitude of the forcing is relatively representative of the maximum observed El Niño in austral summer, but is considerably stronger than recently observed El Niños in austral winter. Despite the idealized nature of the experiments, the design allows us to isolate the teleconnection in different seasons and to distinguish its variation across a consistent set of El Niño amplitudes. The approach therefore overcomes some of the limitations of the observational record in which each El Niño event has a different structure, magnitude and temporal evolution, thereby rendering it difficult to identify variations in the teleconnection to the ASR and the mechanisms that underpin such variations. All anomalies in this study refer to the mean difference between the El Niño and the control experiment unless otherwise specified.

### 2.2 Wave diagnostics

#### 2.2.1 Rossby wave source

Following Sardeshmukh and Hoskins (1988), the Rossby wave source (RWS; S) can be defined as

(2) $$S=-\zeta D-v_{\chi }\nabla \zeta_{x}-v_{\chi_{y}}\nabla \zeta_{y},$$

where ζ is the absolute vorticity, D is the divergence of the horizontal wind, $v_{x}$ is the zonal component of the divergent wind and $v_{y}$ is the meridional component of the divergent wind. The first term (ζD) represents the rate of change of vorticity due to vortex stretching and the second ($v_{x}\nabla \zeta_{x}$) and third ($v_{y}\nabla \zeta_{y}$) terms represent the rate of change of vorticity due to vorticity advection by the zonal and meridional components of the divergent wind, respectively. We use the RWS to identify where El Niño forcing alters the wave source regions that are important for teleconnections to high latitudes.

#### 2.2.2 Rossby wave ray tracing

Rossby wave ray tracing provides insights to how the background atmospheric state impacts the propagation of a linear Rossby wave (Hoskins and Karoly, 1981; Karoly and Hoskins, 1982; Li *et al*., 2015a). The theory for ray tracing shown below follows Hoskins and Karoly (1981), Karoly and Hoskins (1982) and Hoskins and Ambrizzi (1993), and is as described in Yiu and Maycock (2019). Note that, while ray tracing is built on several simplifying assumptions (discussed below), linear wave theory has been successfully applied in many teleconnection studies  (e.g., Hoskins and Karoly, 1981; Karoly and Hoskins, 1982; Li et al., 2015a). The equations are presented in Cartesian coordinates. We start with the simplest case of a linear, barotropic Rossby wave dispersion relation with no background meridional flow

(3) $$\omega =Uk-\frac{\beta^{*}k}{K^{2}},$$

where ω is the frequency, u is the zonal wind, $\beta^{*}$ is the meridional gradient of absolute vorticity, K is the total wavenumber ($K=\sqrt{l^{2}+k^{2}}$), k is the zonal wavenumber and l is the meridional wavenumber. Note that the meridional gradient of absolute vorticity ($\beta^{*}$) can be expressed as $\beta^{*}=\beta -u_{y}$, where β is the meridional gradient of planetary vorticity and $u_{yy}$ is the second derivative of the zonal wind with respect to latitude.

As our study examines signals on seasonal time‐scales, which is longer than the typical Rossby wave propagation time‐scale, we consider the case of stationary waves where ω=0. In this case Equation (3) can be rewritten as

(4) $$K^{2}=\frac{\beta^{*}}{U}.$$

Note that the wave train evolves according to the propagation of wave energy (given by the group velocity). The group velocity of the waves is

(5) $$c_{gx}=\frac{\partial \omega }{\partial k}=\frac{2\beta^{*}k^{2}}{K^{2}},$$

(6) $$c_{gy}=\frac{\partial \omega }{\partial l}=\frac{2\beta^{*}kl}{K^{2}},$$

where $c_{gx}$and $c_{gy}$ are the group velocities in the x, y directions. Thus the direction of propagation of the wave front can be found by dividing Equation (5) by Equation (6) to give

(7) $$\frac{dx}{dy}=\frac{c_{gx}}{c_{gy}}=\frac{k}{l}.$$

$K=\sqrt{l^{2}+k^{2}}$ and Equation (4) can be used to rewrite Equation (7) to give

(8) $$\frac{dx}{dy}=\frac{k}{\sqrt{\frac{\beta^{*}}{U}-k^{2}}}.$$

The ray angle ($\tan^{-1}$(dy/dx)) can be computed for any given k. Following Li *et al*. (2015a) and Yiu and Maycock (2019), ray tracing is performed using k=3. Note that sensitivity tests using k=1,2,4 and 5 showed only small differences in the paths of the rays. As our work is concerned with wave propagation into the Southern Hemisphere, we ignore any northward propagating waves emanating from Rossby wave source regions. Additionally, following Li *et al*. (2015a) and Yiu and Maycock (2019), a 2D Gaussian filter with radius of 15° is applied to the $\beta^{*}$ and u fields prior to the ray calculation. This is because the Rossby waves at k=3 are large compared to the climate model gridscale and hence will not be affected by small‐scale features in the background absolute vorticity and wind fields.

The rays are initialized within a 40° × 10° longitude‐latitude box centred on the maximum RWS anomaly in the southern subtropics in each experiment. A ray is initialized every 2° latitude and 4° longitude within the box giving a total of 50 rays for each experiment. At any point during its propagation, the wave can either propagate, reflect or evanesce. The wave is reflected when $\beta^{*}/u<k^{2}$ (i.e., when the denominator in Equation (8) is imaginary). The waves are evanescent when u<0 (i.e., easterly winds). In other cases, the ray will propagate in the direction given by Equation (8). Ray tracing is performed using climatological seasonal mean fields, but a test was conducted in which ray tracing was performed for every individual season before summation. The results were found to be similar between these approaches, so for simplicity we adopt the former method.

There are several assumptions and simplifications that are used in the derivation of Equation (8). Firstly, Equation (3) is only valid for purely zonal flow. However, for realistic meridional flows the equation can be used as an approximation as long as the zonal gradients of the absolute vorticity are small relative to the meridional gradient of absolute vorticity. This is true in most extratropical cases as the background flow is organized into zonal jets. Secondly, the WKBJ approximation is used, which assumes that variations in the background flow are slow relative to the variations associated with the wave. As discussed in Hoskins and Karoly (1981) and Li *et al*. (2015a), this assumption holds well for small‐scale waves, but may not be as applicable for planetary‐scale waves.

#### 2.2.3 Wave activity flux

In addition to the measure of wave propagation provided by the Rossby wave ray tracing, we also compute the 2D wave activity flux following Plumb (1985). This combines information about both wave sources and wave propagation and gives an overall quantitative picture of wave pseudomomentum fluxes.

Following Plumb (1985), the 2D horizontal wave activity flux is given by

(9) $$F_{s}=p\cos(\phi )\left(\begin{array}{c} v^{\prime 2}-\frac{1}{2\Omega a\sin2\phi }\frac{\partial (v^{\prime }\Phi^{\prime })}{\partial \lambda }\\ -u^{\prime }v^{\prime }+\frac{1}{2\Omega a\sin2\phi }\frac{\partial (u^{\prime }\Phi^{\prime })}{\partial \lambda } \end{array}\right),$$

where primes denote deviations from the zonal mean, p is pressure divided by 1000 hPa, u is zonal wind, v is the meridional wind, Ω is the Earth's rotation rate, a is the radius of the Earth, ϕ is latitude, Φ is geopotential and λ is longitude. The wave activity flux is computed for individual months in the experiments and then averaged to produce seasonal mean fluxes.

## 3 Results

**Figure 2:**
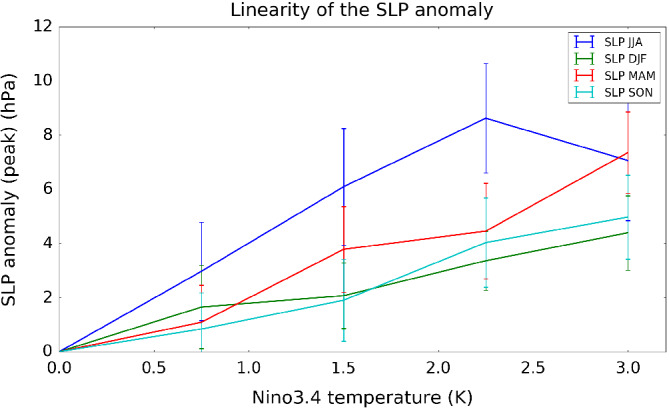
The peak anomaly in seasonal mean sea level pressure (hPa) in the Amundsen Sea region in the four El Niño experiments (0.75, 1.5, 2.25, 3.0 K). Error bars show ±2 standard errors calculated from the interannual variability in the experiments [Colour figure can be viewed at wileyonlinelibrary.com]

**Figure 3:**
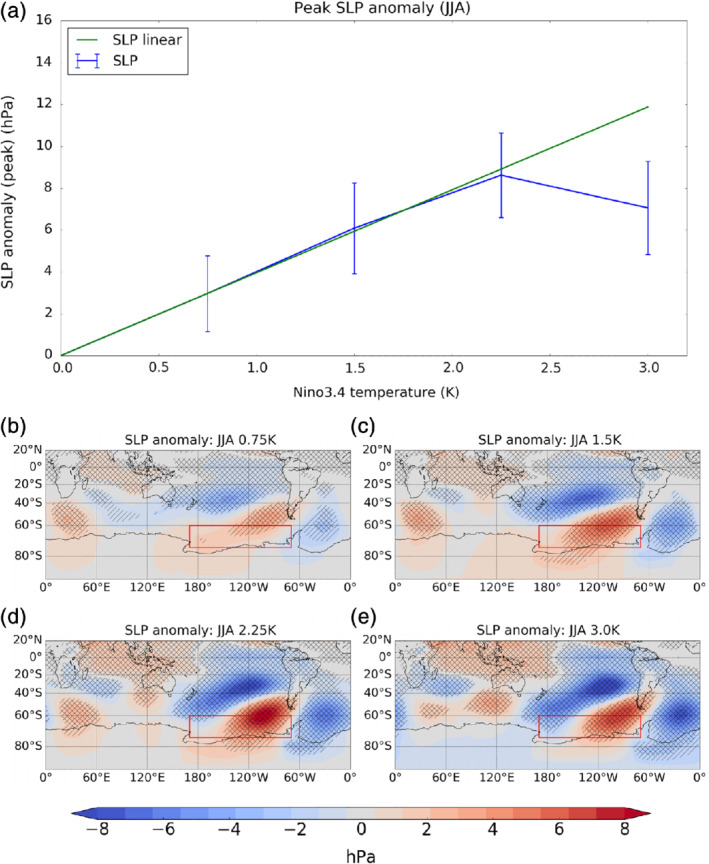
(a) The peak sea level pressure anomaly (hPa) in the Amundsen Sea region for JJA as a function of El Niño amplitude. The blue line connects the mean anomalies from the model experiments. Error bars show ±2 standard errors. The green line shows the anomaly in the 0.75 K experiment linearly extrapolated as a function of El Niño amplitude. (b–e) Maps of SLP anomalies (shading) in JJA for 0.75, 1.5, 2.25 and 3 K El Niño experiments, respectively. Single hatching shows regions of 95% significance while double hatching shows 99% significance [Colour figure can be viewed at wileyonlinelibrary.com]

**Figure 4:**
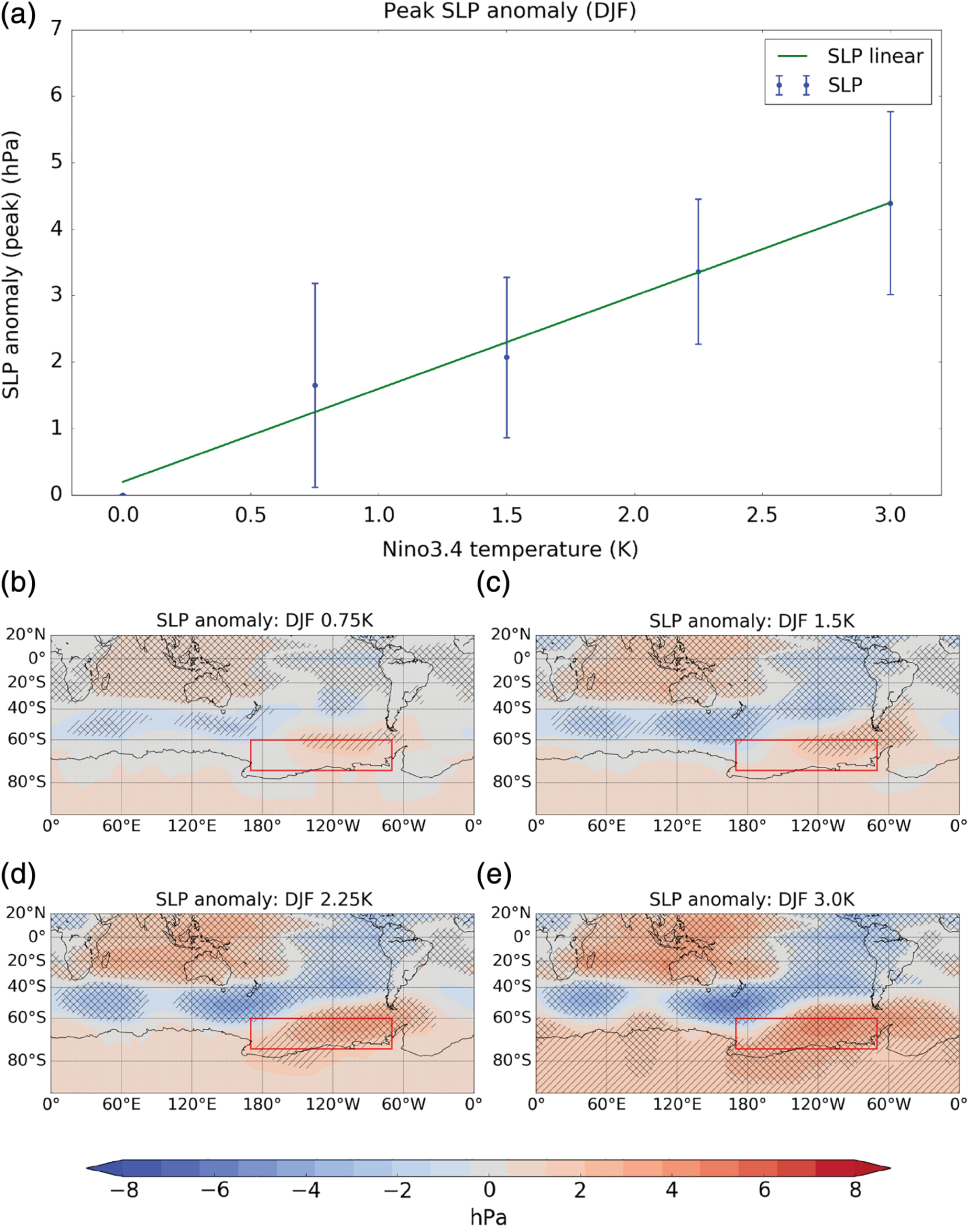
(a) The peak sea level pressure (hPa) anomaly in the Amundsen Sea region for DJF as a function of El Niño amplitude. The blue line shows model simulations, and the green shows a linear extrapolation as in Figure 3a. Error bars show ±2 standard errors. (b–e) Maps of SLP anomalies (shading) in DJF for 0.75, 1.5, 2.25 and 3 K El Niño experiments, respectively. Single hatching shows regions of 95% significance while double hatching shows 99% significance [Colour figure can be viewed at wileyonlinelibrary.com]

**Figure 5:**
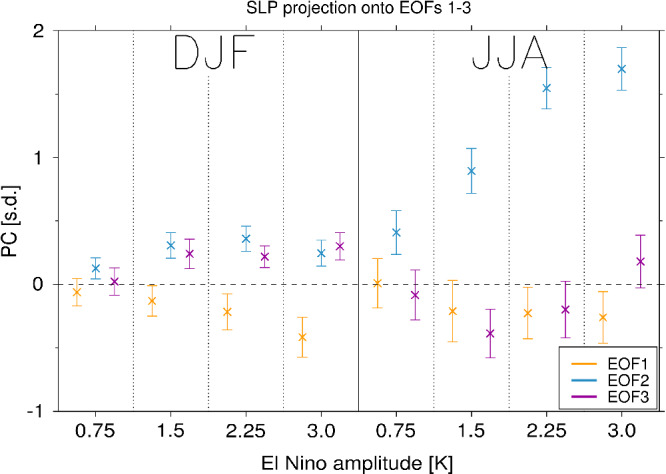
Projection of the extratropical sea level pressure response (30°S–90°S) onto EOFs 1–3 as a function of El Niño amplitude for (left) DJF and (right) JJA seasons. Whiskers show ±2 standard errors based on the interannual variability [Colour figure can be viewed at wileyonlinelibrary.com]

### 3.1 Seasonal response and projection onto leading modes

Figure 2 shows the local maximum sea level pressure (SLP) anomaly within the ASR (170°E–290°E, 60°S–75°S) in all four seasons (DJF, MAM, JJA, SON) as a function of El Niño amplitude. Consistent with earlier studies (Bertler *et al*., 2004; Turner, 2004; Jin and Kirtman, 2009; Yiu and Maycock, 2019), the SLP response to El Niño is consistently largest in JJA (blue line) compared to other seasons, while in DJF (green line) and SON (cyan line) the SLP response is weakest, especially under larger El Niño forcing. Only the JJA SLP response shows an indication of nonlinear behaviour with increasing El Niño amplitude, with the response in the 3 K experiment being on average weaker than in the 2.25 K case. Similar behaviour is found for the area‐averaged SLP anomaly in the ASR (Supporting Information Figure S2). This is further demonstrated in Figure 3a, which shows the maximum ASR SLP anomaly in JJA (blue line) alongside a linear extrapolation of the SLP response in the 0.75 K El Niño experiment to an amplitude of 3 K (green line). The maximum SLP anomaly in JJA (±2 standard errors) is 3.0 ± 0.7 hPa, 6.1 ± 0.8 hPa, 8.6 ± 0.7 hPa and 7.0 ± 0.7 hPa for the 0.75 K, 1.5 K, 2.25 K, 3.0 K El Niño experiments, respectively. The amplitude of the peak SLP anomaly in the ASR therefore increases by ∼3 hPa per 0.75 K for El Niño amplitudes up to 2.25 K. However, in the 3.0 K El Niño experiment, the peak SLP anomaly decreases by ∼1.6 hPa compared to the 2.25 K case despite the larger SST forcing. Observation‐based data have a much sparser sampling of El Niño magnitudes, which makes it difficult to extract a robust signal of how the teleconnection varies with forcing amplitude. Nevertheless, across all years since 1870, a seasonal variation in the relationship between the ASR‐averaged SLP anomaly and the Niño 3.4 anomaly is evident, with the magnitude being ∼60% larger in JJA than in DJF (Supporting Information Figure S3).

Figure 3b–e shows maps of the Southern Hemisphere SLP anomalies in JJA in the four El Niño experiments. While the gross spatial features of the SLP responses are similar between the experiments, all resembling the PSA pattern (Karoly, 1989; Yiu and Maycock, 2019), there are some structural differences. For example, the negative pressure anomaly at around 30°S and 180°W–100°W appears to elongate and shift eastward as El Niño magnitude increases. In the 0.75 K El Niño  experiment, the centre of the negative SLP anomaly is near 150°W while it is around 110°W in the 3.0 K experiment. This point will be revisited later in Section 3.2. A further structural change in the response is that the sign of the SLP anomaly in the Ross Sea changes from positive to negative with increasing El Niño amplitude. This may be important for understanding the relationship between the ASL and climate further west in the Ross Sea sector (Coggins and McDonald, 2015).

For comparison, Figure 4 shows the same as Figure 3 but for the DJF season. In Figure 4a the modelled peak SLP response in the ASR across all experiments is well described by a linear extrapolation of the response to the weakest 0.75 K El Niño to larger amplitudes. The maps of Southern Hemisphere SLP changes in DJF in Figure 4b–e show a more zonally symmetric pattern than in JJA, but with some local enhancement in the ASR. The patterns of SLP anomalies to El Niño in both seasons compare well with composite responses derived from reanalysis data (Yiu and Maycock, 2019).

**Figure 6:**
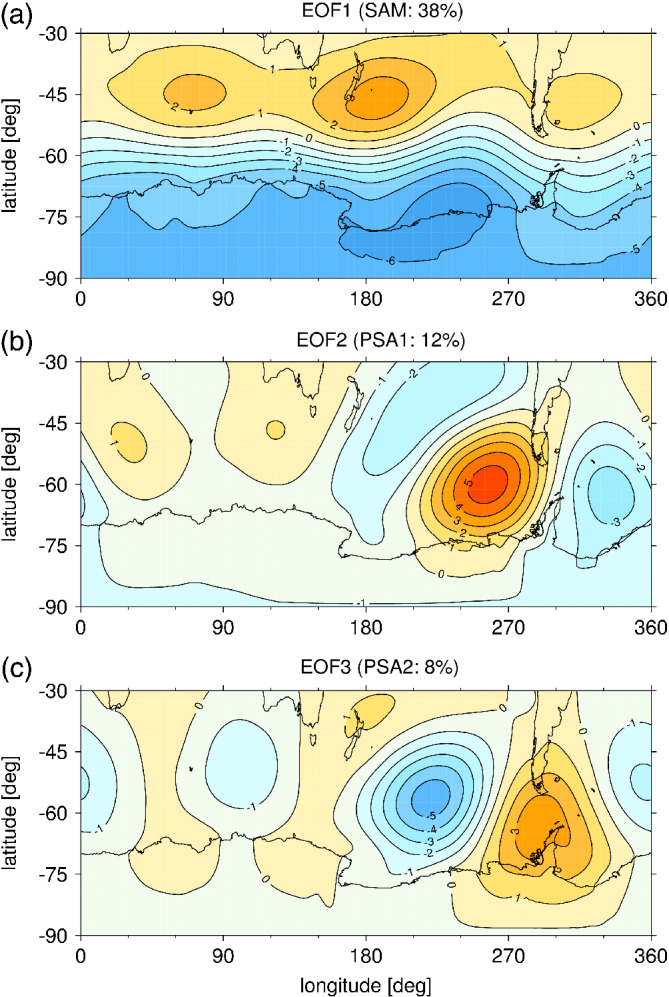
EOFs 1‐3 of monthly mean Southern Hemisphere extratropical (30–90°S) sea level pressure in HadGEM3‐A. Each pattern is plotted to correspond to a positive principal component (PC). The percentages in the headers denote the variance explained by each pattern [Colour figure can be viewed at wileyonlinelibrary.com]

Given the pronounced differences in the pattern of SLP response to El Niño between austral winter and summer seasons, Figure 5 shows the principal components (PC) that describe the projection of the seasonal mean SLP response for each El Niño amplitude onto the three leading Empirical Orthogonal Functions (EOFs) of monthly extratropical (ϕ≤ –30°) SLP. The SLP patterns corresponding to EOFs 1–3 are shown in Figure 6. EOF1 represents the SAM, EOF2 represents the PSA pattern and EOF3 represents the South Pacific wave (SPW) pattern (Mo, 2000). In DJF, the SLP response to El Niño projects onto a negative SAM (EOF1) and positive PSA (EOF2) and SPW (EOF3) patterns (left, Figure 5). The projection onto a negative SAM in DJF increases monotonically with El Niño amplitude, while for PC2 and PC3 there is an initial increase in amplitude between 0.75 K and 1.5 K El Niño, but no further change under stronger forcing. This reflects that in austral summer the SLP response to El Niño is more zonally symmetric than in austral winter.

In JJA, the strongest projection across all El Niño amplitudes is onto EOF2 (right, Figure 5), which is consistent with the pronounced tropical–extratropical wave train pattern seen in Figure 3. The amplitude of PC2 increases monotonically and approximately linearly for El Niño amplitudes up to 2.25 K. However, there is no statistically significant difference in the magnitude of PC2 between 2.25 K and 3 K El Niño forcing. As EOF2 describes a tropical–extratropical Rossby wave train, this suggests the onset of a saturation effect in either the wave source and/or wave propagation characteristics. This will be further investigated in Section 3.2. There is no significant projection onto the SAM in JJA in the 0.75 K El Niño experiment, but in the three stronger El Niño experiments there is a weak projection onto a negative SAM with an amplitude comparable to that in DJF in the 2.25 K experiment. The SLP projection onto EOF3 in JJA shows a nonlinear behaviour. PC3 is increasingly negative up to 1.5 K El Niño, but then increases for larger El Niño forcing so that it becomes positive in the 3 K experiment. The reversal in sign of PC3 and the sub‐linear behaviour of PC2 with increasing El Niño amplitude explain the nonlinear behaviour of the peak ASR SLP seen in Figure 3. Note there are residuals of the SLP response that are not explained by EOFs 1–3 (Supporting Information Figure S4); while the residuals show anomalies in some parts of the southern extratropics, in the ASR these are generally small compared to the total responses seen in Figures 3 and 4.

In summary, Figure 3 shows that the ASR response in austral winter is approximately linear for imposed El Niños within historically observed amplitudes for this season (up to ∼1.5 K), but the response becomes sub‐linear for stronger forcing (>2.25 K in JJA). This is manifested as changes in the projection of the extratropical SLP response onto the PSA (EOF2) and SPW (EOF3) patterns in particular. In contrast, the amplitude of the ASR SLP anomalies in austral summer (DJF) increases approximately linearly over the range of El Niño amplitudes considered (Figure 4), and this is mainly captured by an increase in the SAM (EOF1). We now investigate the dynamical processes that underpin the nonlinearity of the ASR response in austral winter in the 3.0 K El Niño experiment.

### 3.2 Investigating the nonlinear Amundsen Sea region SLP response in austral winter (JJA)

To understand why the magnitude of the ASR SLP anomaly in JJA in the 3.0 K El Niño experiment is weaker than in the 2.25 K experiment, it is necessary to look at both the RWS and Rossby wave propagation characteristics.

**Figure 7:**
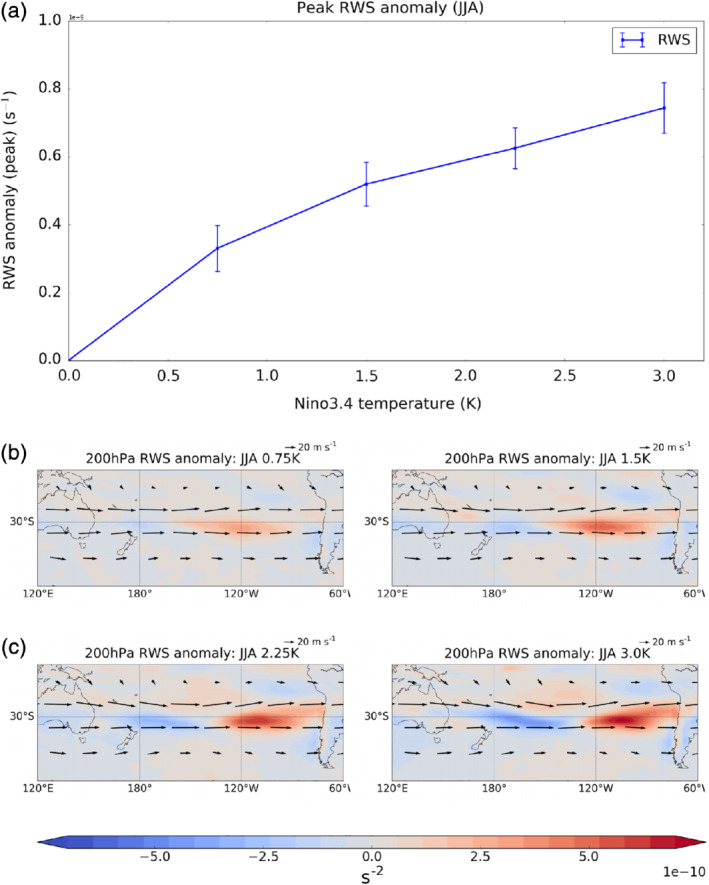
(a) Maximum Rossby wave source anomaly (s${}^{-2}$) in the South Pacific sector (15°S–40°S, 180°E–300°E) for JJA as a function of El Niño amplitude. Error bars show ±2 standard errors. (b–e) Maps of the Rossby wave source anomaly (shading) and absolute wind vectors for JJA in the different El Niño experiments [Colour figure can be viewed at wileyonlinelibrary.com]

We first examine the Rossby wave characteristics. Figure 7a shows the peak RWS anomaly at 200 hPa (calculated using Equation (2)) in the South Pacific sector (25–35°S and 180°E–270°E). This region encompasses the location of the maximum RWS in all the experiments. The maximum RWS anomaly in the South Pacific increases monotonically with El Niño amplitude. The maps of RWS anomalies in Figure 7b–e show that, as El Niño strength increases, the dipole RWS anomaly between the east and west Pacific around 30°S–35°S shifts eastward and increases in amplitude. The longitude of the maximum positive RWS anomaly moves eastward by around 15° longitude between the 0.75 K and 3.0 K experiments. Yiu and Maycock (2019) showed that in the 1.5 K El Niño experiment, the change in RWS in austral winter is dominated by the vortex stretching term (term 1 in Equation (2)) due to the changes in upper tropospheric divergence. This appears consistent in all the experiments as the patterns of anomalous RWS (Figure 7b–e) closely resemble the anomalous upper tropospheric divergence (Supporting Information Figure S5). Hence the intensification of tropical convection and the associated eastward shift of the region of strong anomalous divergence near the jet exit region is key for the anomalous RWS under increasing El Niño forcing. However, the peak RWS anomaly does not decrease in the 3 K El Niño experiment relative to the 2.25 K case, and thus the amplitude of the anomalous RWS alone is unlikely to explain the reduced ASR SLP response shown in Figure 3a.

**Figure 8:**
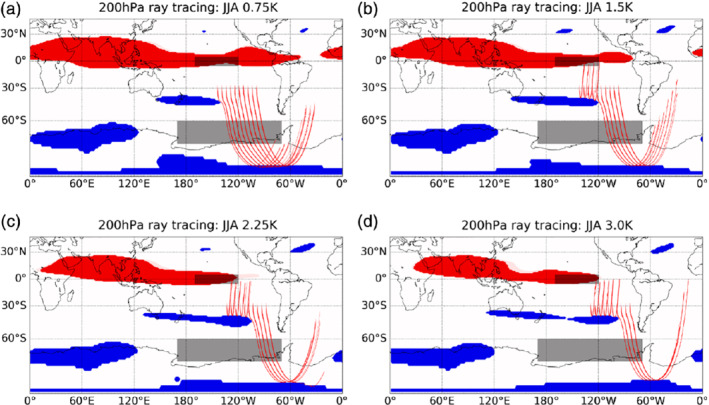
Red lines show Rossby wave ray traces (k=3) in JJA at 200 hPa for the four El Niño experiments. Blue shading shows regions of wave reflection and red shading shows regions of wave evanescence. Rays are initialized 
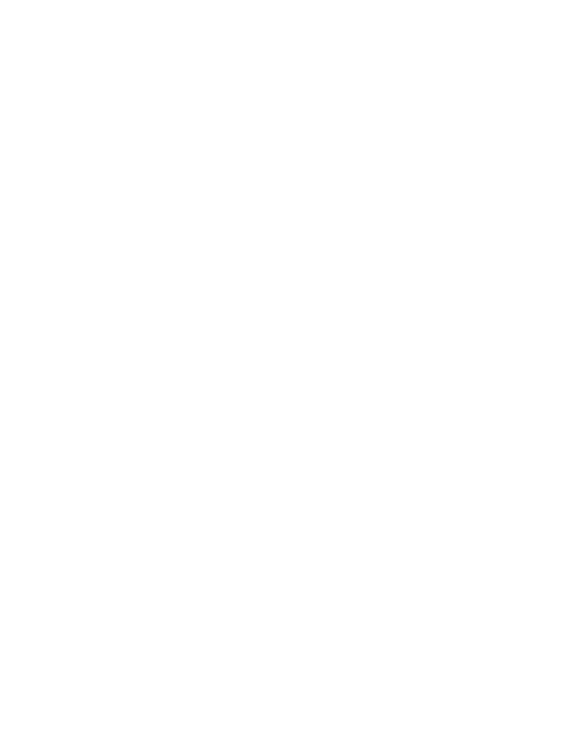
 centred at the approximate location of the peak positive Rossby wave source anomaly in Figure 7 [Colour figure can be viewed at wileyonlinelibrary.com]

**Figure 9:**
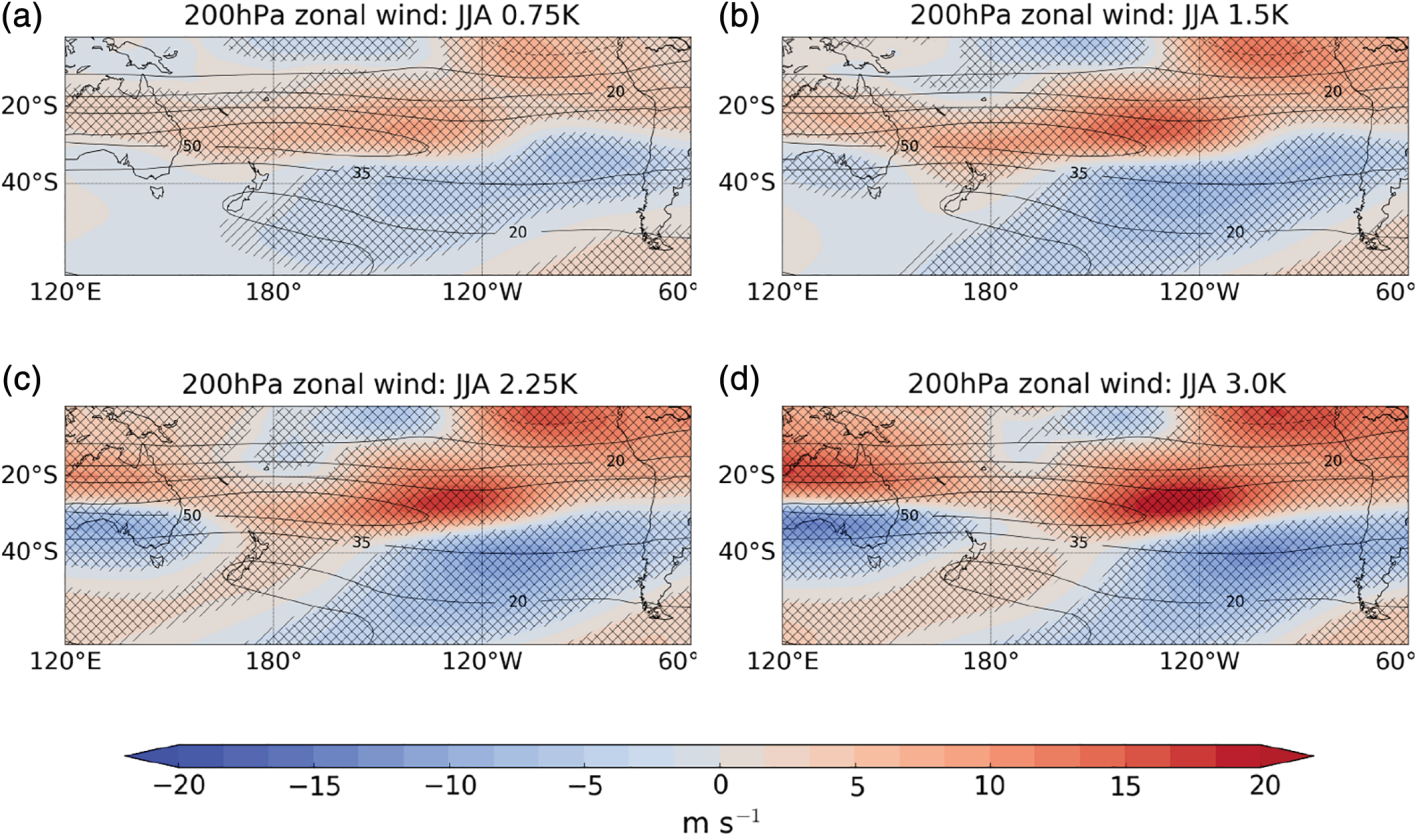
Maps of 200 hPa zonal wind anomalies (shading) for JJA in the South Pacific for the four El Niño experiments. Single hatching shows regions of 95% significance and double hatching shows 99% significance. Contours show the climatology of the control experiment [Colour figure can be viewed at wileyonlinelibrary.com]

To investigate how the shift in RWS region and other changes in background flow influence Rossby wave propagation to high latitudes, Figure 8 shows hypothetical Rossby wave ray traces for the four El Niño experiments calculated using Equation (8) and k=3. For comparison, equivalent calculations for the 1.5 K El Niño experiment using k=2 and k=4 are shown in the Supporting Information Figure S6. The red shading in Figure 8 denotes regions where waves are terminated and blue shows regions of wave reflection. Note that the ray tracing only provides a qualitative measure of Rossby wave propagation under certain simplifying assumptions and does not correspond to a direct measure of wave energy. Nevertheless, it does provide insight to whether conditions are favourable for wave propagation in the different El Niño experiments (Li *et al*., 2015b; Yiu and Maycock, 2019).

Figure 8 shows that as El Niño strength increases, a wave reflection surface that is located initially in the west Pacific around 40°S extends eastward, such that when the El Niño perturbation reaches 3.0 K it spans much of the South Pacific sector from 110°E to 100°W. As shown in Equation (8), the conditions for wave reflection are strongly related to $\beta^{*}=\beta -u_{yy}$. In the case of constant latitude (i.e., constant β), changes to $\beta^{*}$ will be determined by $u_{yy}$. The background upper tropospheric zonal wind is therefore important for the poleward propagation of Rossby waves emanating from the Tropics (e.g., Hoskins and Karoly, 1981; Sardeshmukh and Hoskins, 1988).

Figure 9 shows maps of the 200 hPa zonal wind anomalies in the South Pacific sector in the four El Niño experiments. The solid contours show the climatological zonal wind in the control experiment and the shading shows the anomalies under El Niño. The subtropical jet over the South Pacific strengthens, extends downstream and contracts equatorward in response to El Niño (Lu *et al*., 2008). These structural changes in the wind field enable us to understand the wave reflection behaviour as they are associated with changes in $u_{yy}$ and hence in $\beta^{*}$. Figure 10b–e show maps of $u_{yy}$ in the different El Niño experiments. The region of high $u_{yy}$ values associated with the subtropical jet extends eastward under increasing El Niño forcing. We calculate using Equation 8 that within this range of latitudes (∼30°S–45°S), regions where $u_{yy}$ ≥ 2.7 m^−1^ s^−1^ will be associated with Rossby wave reflection, while rays will propagate through regions where $u_{yy}$ < 2.7 m^−1^ s^−1^. Note that this threshold is approximate as it ignores the contribution from changes in β to $\beta^{*}$ and thus does not account for the small latitudinal variations in the location of the wave reflection surface. Nevertheless, the propagation and reflection of the ray traces plotted in Figure 8b–e closely coincide with this approximate threshold.

Figure 10a shows the peak $u_{yy}$ value between 30°S and 45°S as a function of longitude in the control and four El Niño experiments. The horizontal dotted lines show the most eastward location where $u_{yy}$ drops below the estimated threshold to permit wave propagation; this is at ∼215°E, 230°E, 240°E, 250°E and 265°E for the control and four El Niño experiments, respectively. Hence the eastward extent of the region in the South Pacific where Rossby wave reflection occurs increases with El Niño amplitude. Of course, a wave reflection zone ending at a certain point does not mean that all Rossby waves will be blocked at that longitude. This is because, as seen in the ray traces in Figure 8, the waves are also propagating longitudinally. However, for the case considered here, the latitudinal movement is usually much greater than longitudinal movement.

Recall that in Figure 7 the maximum RWS anomaly was also found to move eastward with increasing El Niño amplitude. To quantify this further, Figure 11a shows the longitude of the maximum 200 hPa RWS anomaly between 15°S and 45°S as a function of El Niño amplitude. In the 0.75 K El Niño experiment, the strongest RWS anomaly lies to the east of the region where conditions favour wave reflection (red line, Figure 11a). However, in the 3 K El Niño experiment, the maximum RWS anomaly is located within the region where wave reflection is expected to occur (yellow line Figure 11a). Hence while both the anomalous RWS and the wave reflection surface shift eastward with increasing El Niño amplitude, the latter moves relatively more. This is seen more clearly in Figure 11b, which shows the eastward extent of the wave reflection surface (green line), as defined by $u_{yy}$ < 2.7 m^−1^ s^−1^, and the longitude of the peak 200 hPa RWS anomaly (blue line) for the different El Niño experiments. The former shows a larger relative change than the latter as El Niño amplitude increases. The anomalous RWS is predominantly a consequence of the shifting upper‐level divergence (Supporting Information Figure S5) while the shifting of the wave reflection surface is a consequence of the changes in zonal wind curvature, which is controlled by both the strengthening of westerlies on the equatorward side of the subtropical jet and the weakening of westerlies on the poleward side. Hence, while related, we do not expect these two factors to exhibit identical behaviour. Also shown in Figure 11b (red line) is the longitude of the maximum SLP anomaly in the ASR sector. The longitude of the peak ASR SLP anomaly closely tracks the RWS anomaly, demonstrating the changing structure of the SLP response is primarily associated with a shift in the wave train with the anomalous RWS. As the wave train moves eastward, its projection onto the SPW pattern (EOF3) eventually changes sign (right, Figure 5).

The analysis presented so far shows the overall effect in the 3 K El Niño experiment is for wave propagation to high latitudes in austral winter to be relatively more restricted than in the 2.25 K case. To further test this, we lastly analyse a quantitative measure of wave activity flux as defined in Equation 9. Figure 12b–e show maps of 200 hPa wave flux anomalies in JJA. There is an increase in the anomalous poleward propagation of wave energy in the subtropics and midlatitudes as El Niño amplitude increases. There is also a marked increase in anomalous wave flux in the ASR for El Niño amplitudes up to 2.25 K. However, there is no clear increase in wave fluxes in the ASR between 2.25 K and 3 K. Though we cannot quantitatively compare the different methods, this is broadly consistent with our previous findings based on the ray tracing. It is interesting to attempt to quantify the total wave activity flux anomalies in the region of the South Pacific where wave propagation is possible. To do so, we plot the meridional wave flux anomalies at 45°S averaged over 180°E–270°E as a function of El Niño amplitude (Figure 12a). This represents the component of wave pseudomomentum propagating poleward towards the ASR. Similarly to what was found for the SLP anomaly in Figure 3, the meridional wave flux shows an almost linear behaviour for El Niño forcing up to 2.25 K, but deviates to a sub‐linear relationship in the 3.0 K El Niño experiment. This picture is consistent with the previous results discussed in this section.

## 4 Conclusions

The El Niño Southern Oscillation is an important driver of interannual climate variability in West Antarctica. This study examines how the El Niño teleconnection to the Amundsen Sea region (ASR) varies as a function of El Niño amplitude. We analyse idealized experiments performed with the HadGEM3‐A climate model which impose an East Pacific El Niño SST anomaly corresponding to a Niño 3.4 anomaly of 0.75, 1.5, 2.25 and 3 K. In austral summer (DJF), the teleconnection to the ASR, as measured by the local SLP anomaly, is found to be approximately linear for all El Niño amplitudes explored and projects onto a negative phase of the SAM. In contrast, in austral winter (JJA) the teleconnection behaves approximately linearly for El Niño amplitudes up to 2.25 K, but for a stronger El Niño forcing of 3 K the teleconnection in the ASR weakens despite the larger SST forcing. This is associated with a switch from a negative to positive projection of the SLP response onto the South Pacific wave pattern (EOF3) and a relatively smaller increase in the projection onto PSA (EOF2). To investigate the causes of this behaviour, the changes in Rossby wave source and Rossby wave propagation under the different El Niño conditions were analysed. This revealed that the region of strongest RWS in the South Pacific moves progressively eastward with increasing El Niño amplitude, causing an eastward shift in the Rossby wave train and a change in the sign of the SLP anomalies in the Ross Sea. However, the wave reflection surface associated with curvature in the upper tropospheric zonal winds also moves eastward but at a faster rate. This means that for stronger El Niños the relative propensity for wave propagation to high latitudes is diminished and the teleconnection actually weakens despite the El Niño forcing being larger. We note that the amplitude at which this effect occurs in our experiments is larger than observed El Niños in austral winter and thus, for the purposes of present‐day observation‐based studies, a linear approximation for the El Niño teleconnection in both austral winter and summer is likely to be reasonable.

## Supporting information

Figure S1: Box plots of seasonal Nino 3.4 index from HadISST1 for 1870–2018. Data have been detrended.
Figure S2: Scatterplot of SLP anomalies in the Amundsen Sea region against Nino 3.4 index for (a) DJF and (b) JJA seasons during 1870–2018. The red line shows a linear regression with the gradient in the legend.
Figure S3: (a) The sea level pressure [hPa] anomaly (averaged across 50–75°S, 130–80°W) for JJA as a function of El Nino amplitude. Blue line shows model simulations, green shows linear model. Error bars show ±2 standard errors.
Figure S4: Residual sea level pressure [hPa] response in Southern hemisphere not explained by the projection onto EOFs 1–3 for different El Nino amplitudes (columns) in (a–d) DJF and (e–h) JJA.
Figure S5: Anomalous 200 hPa divergence (shading; s^−1^) and absolute 200 hPa wind vectors in the four El Nino experiments.
Figure S6: Linear barotropic Rossby wave ray traces for the 1.5 K El Nino experiment, as in Figure 8b of the main text, but using (top) k = 2 and (bottom) k = 4. Blue shading shows regions of wave reflection and red shading shows regions of wave evanescence. The ASR is marked by the grey box.
Supplementary Information.
