# Supplementary material for: The linearity of the El Niño teleconnection to the Amundsen Sea region
Source: Q J R Meteorol Soc. 2020 Feb 3;146(728):1169–83. doi: 10.1002/qj.3731 (PMC7317404; doi:10.1002/qj.3731)
Supplement: Supplementary file 1 — Figure S1: Box plots of seasonal Nino 3.4 index from HadISST1 for 1870–2018. Data have been detrended. Figure S2: Scatterplot of SLP anomalies in the Amundsen Sea region against Nino 3.4 index for (a) DJF and (b) JJA seasons during 1870–2018. The red line shows a linear regression with the gradient in the legend. Figure S3: (a) The sea level pressure [hPa] anomaly (averaged across 50–75°S, 130–80°W) for JJA as a function of El Nino amplitude. Blue line shows model simulations, green shows linear model. Error bars show ±2 standard errors. Figure S4: Residual sea level pressure [hPa] response in Southern hemisphere not explained by the projection onto EOFs 1–3 for different El Nino amplitudes (columns) in (a–d) DJF and (e–h) JJA. Figure S5: Anomalous 200 hPa divergence (shading; s−1) and absolute 200 hPa wind vectors in the four El Nino experiments. Figure S6: Linear barotropic Rossby wave ray traces for the 1.5 K El Nino experiment, as in Figure 8b of the main text, but using (top) k = 2 and (bottom) k = 4. Blue shading shows regions of wave reflection and red shading shows regions of wave evanescence. The ASR is marked by the grey box. Supplementary Information. [file QJ-146-1169-s001.pdf]

Supplementary Material

Journal: Quarterly Journal of the Royal Meteorological Society

Title: The linearity of the El Niño teleconnection to the Amundsen Sea region

Authors: Yu Yeung Scott Yiu and Amanda C. Maycock

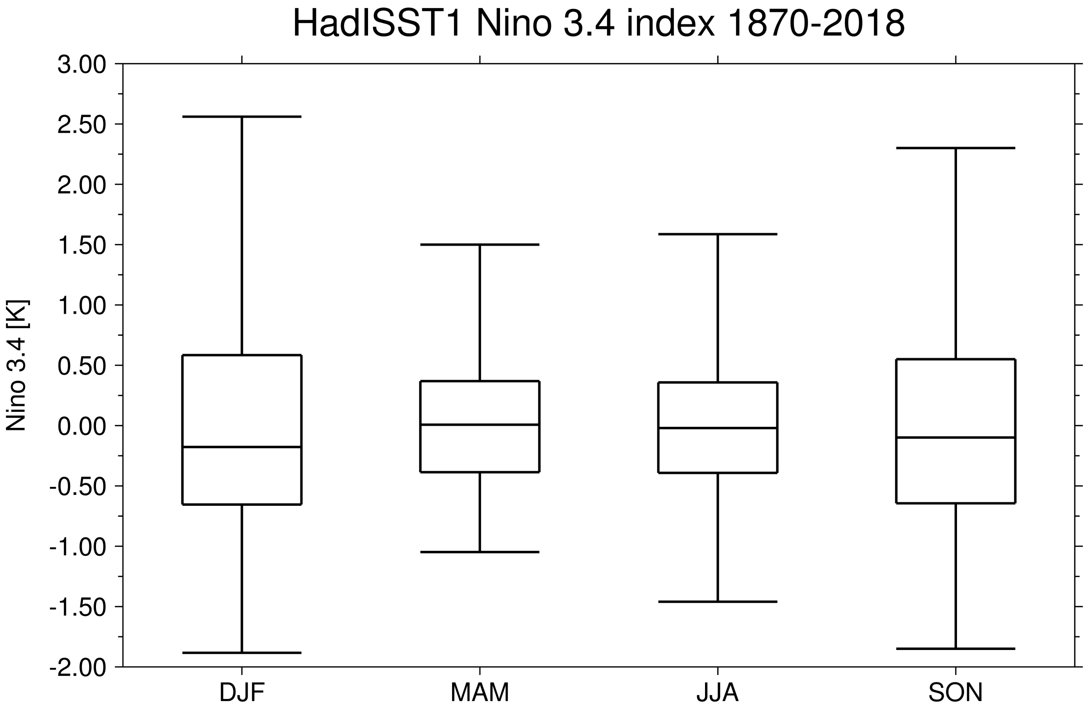

Figure S1: Box plots of seasonal mean Nino 3.4 index [K] from HadISST1 for 1870-2018. Data have been linearly detrended.

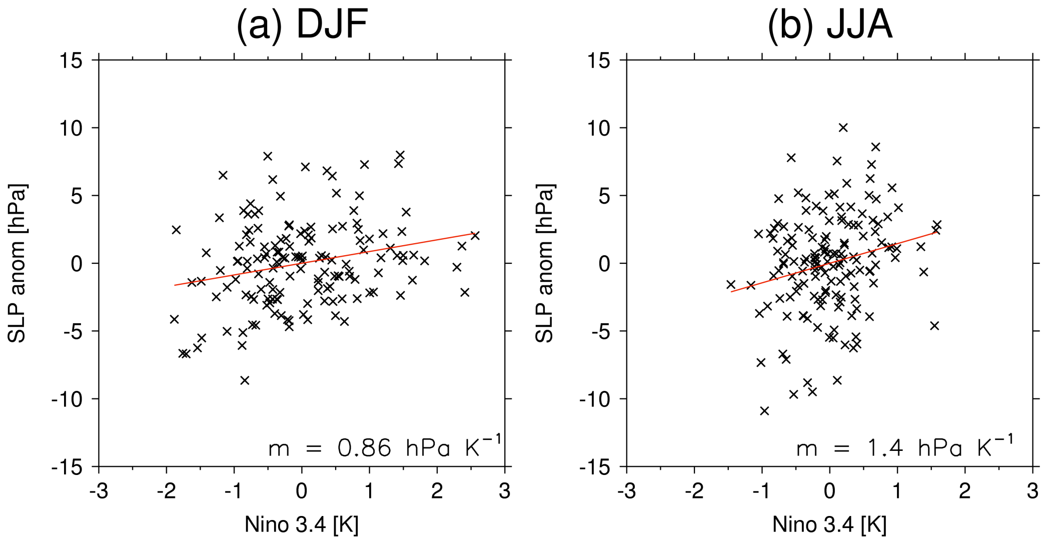

Figure S2: Scatterplot of the observed sea level pressure [hPa] anomaly in the Amundsen Sea region (50–75°S, 130–80°W) against Nino 3.4 index [K] for (a) DJF and (b) JJA seasons during 1870-2018. Each cross shows one year. The red line shows a linear regression with the gradient given in the legend.

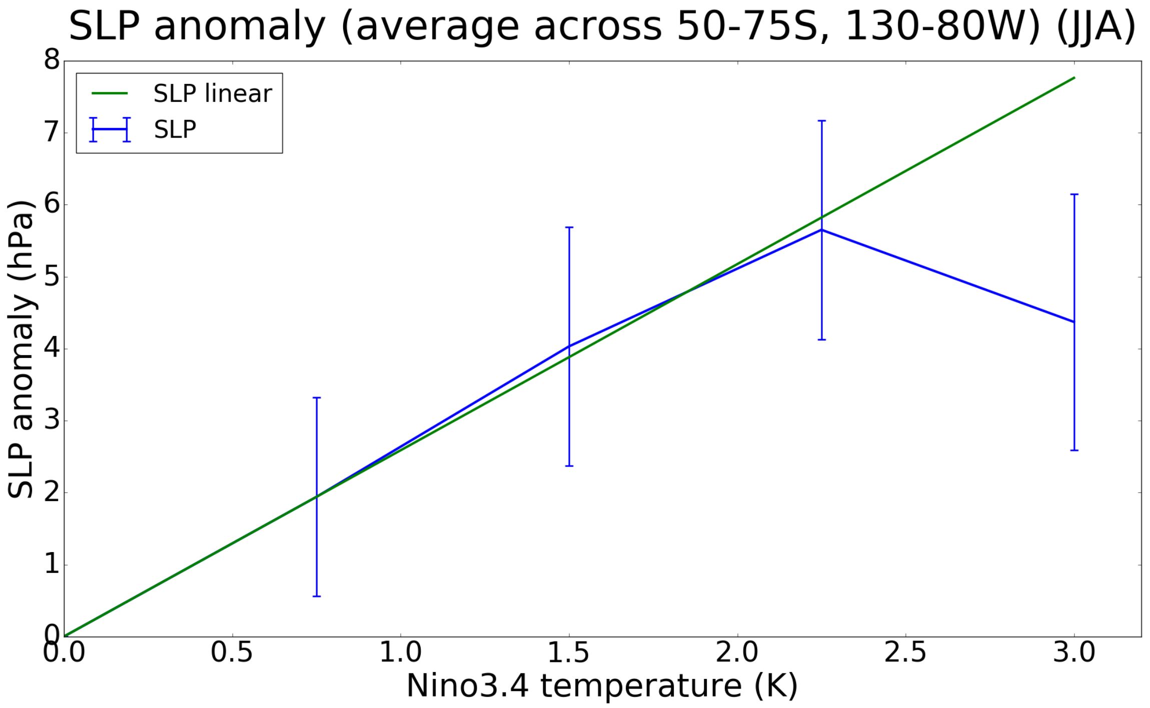

Figure S3: (a) The average sea level pressure [hPa] anomaly in the Amundsen Sea region (50–75°S, 130–80°W) for JJA as a function of El Niño amplitude. Blue line shows model simulations, green shows linear model. Error bars show ±2 standard errors.

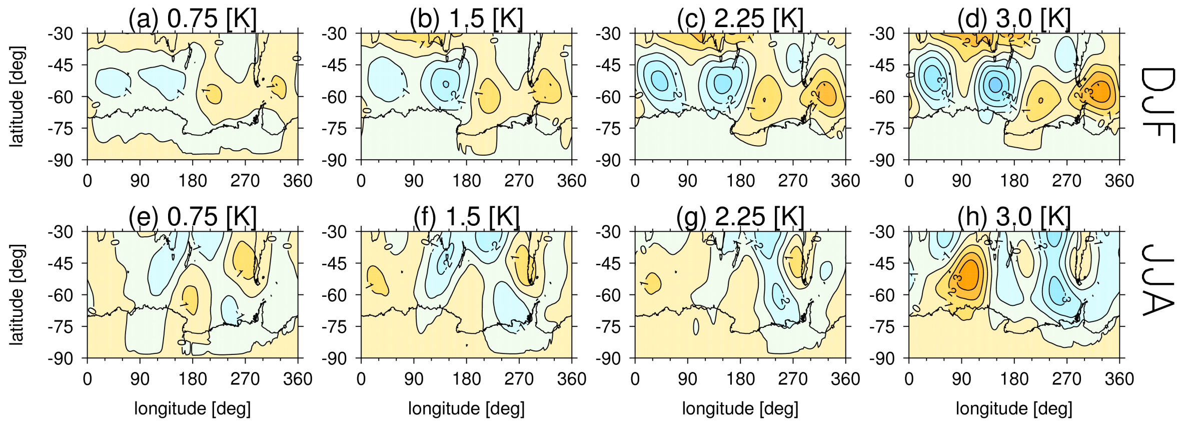

Figure S4: Residual sea level pressure [hPa] response in the Southern hemisphere extra-tropics not explained by the projection onto EOFs 1-3 for different El Niño amplitudes (columns) in (a-d) DJF and (e-h) JJA.

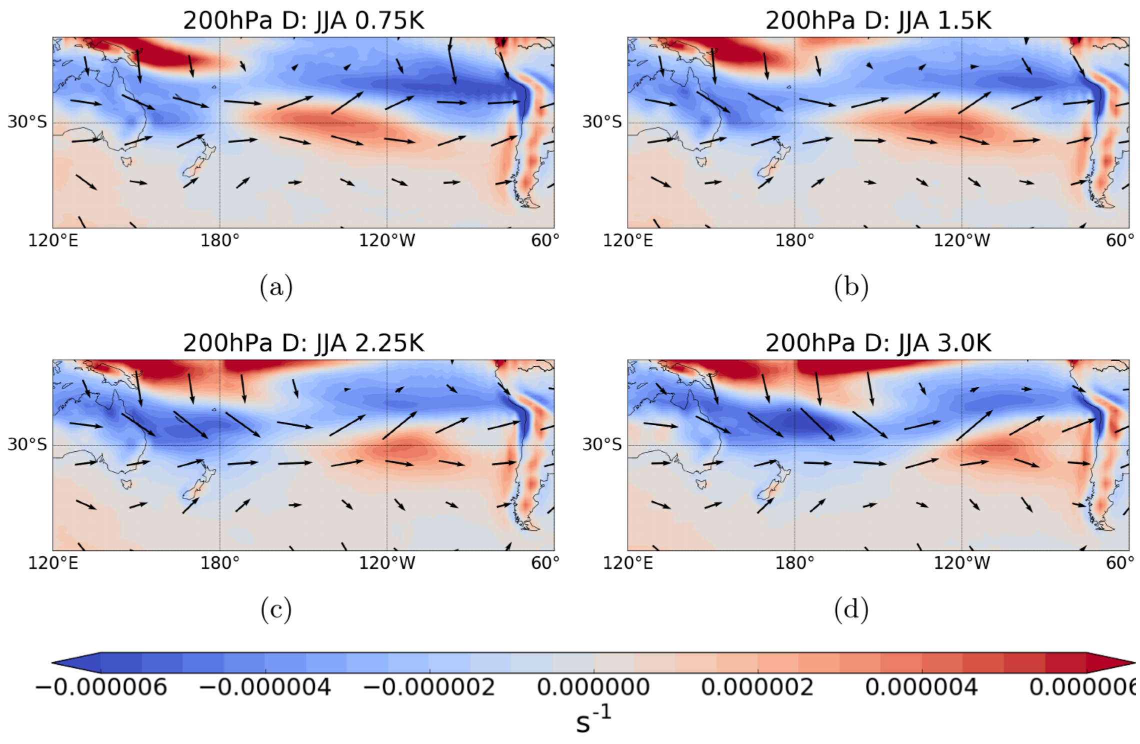

Figure S5: Anomalous 200 hPa divergence (shading; s^{-1}) and absolute 200 hPa wind vectors in the four El Niño experiments.

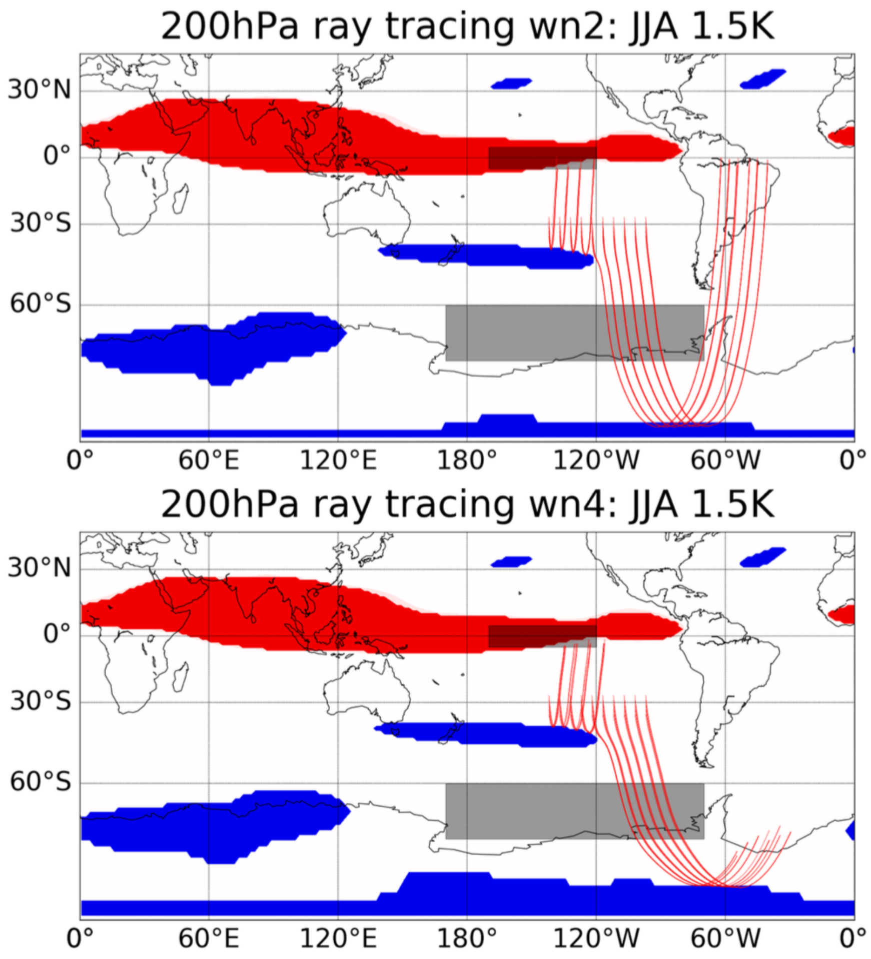

Figure S6: Linear barotropic Rossby wave ray traces for the 1.5 K El Niño experiment, as in Figure 8b of the main text, but using (top) k=2 and (bottom) k=4. Blue shading shows regions of wave reflection and red shading shows regions of wave evanescence. The ASR is marked by the grey box.
